# Assessing the neonatal health policy landscape in low- and middle-income countries: Findings from the 2018 WHO SRMNCAH policy survey

**DOI:** 10.7189/jogh.13.04024

**Published:** 2023-03-03

**Authors:** Meighan Mary, Blerta Maliqi, Elizabeth K Stierman, Martin AJ Dohlsten, Allisyn C Moran, Elizabeth Katwan, Andreea A Creanga

**Affiliations:** 1 Department of International Health, Johns Hopkins Bloomberg School of Public Health, Baltimore, Maryland, USA; 2 International Center for Maternal and Newborn Health, Johns Hopkins Bloomberg School of Public Health, Baltimore, Maryland, USA; 3 Department of Maternal, Newborn, Child, Adolescent Health and Ageing, World Health Organization, Geneva, Switzerland; 4 Department of Gynecology and Obstetrics, Johns Hopkins School of Medicine, Baltimore, Maryland, USA

## Abstract

**Background:**

We aimed to describe the availability of newborn health policies across the continuum of care in low- and middle-income countries (LMICs) and to assess the relationship between the availability of newborn health policies and their achievement of global Sustainable Development Goal and Every Newborn Action Plan (ENAP) neonatal mortality and stillbirth rate targets in 2019.

**Methods:**

We used data from World Health Organization’s 2018-2019 sexual, reproductive, maternal, newborn, child and adolescent health (SRMNCAH) Policy Survey and extracted key newborn health service delivery and cross-cutting health systems policies that align with the WHO health system building blocks. We constructed composite measures to represent packages of newborn health policies for five components along the continuum of care: antenatal care (ANC), childbirth, postnatal care (PNC), essential newborn care (ENC), and management of small and sick newborns (SSNB). We used descriptive analyses to present the differences in the availability of newborn health service delivery policies by World Bank income group in 113 LMICs. We employed logistic regression analysis to assess the relationship between the availability of each composite newborn health policy package and achievement of global neonatal mortality and stillbirth rate targets by 2019.

**Results:**

In 2018, most LMICs had existing policies regarding newborn health across the continuum of care. However, policy specifications varied widely. While the availability of the ANC, childbirth, PNC, and ENC policy packages was not associated with having achieved global NMR targets by 2019, LMICs with existing policy packages on the management of SSNB were 4.4 times more likely to have reached the global NMR target (adjusted odds ratio (aOR) = 4.40; 95% confidence interval (CI) = 1.09-17.79) after controlling for income group and supporting health systems policies.

**Conclusions:**

Given the current trajectory of neonatal mortality in LMICs, there is a dire need for supportive health systems and policy environments for newborn health across the continuum of care. Adoption and implementation of evidence-informed newborn health policies will be a crucial step in putting LMICs on track to meet global newborn and stillbirth targets by 2030.

With less than 10 years to achieve the Sustainable Development Goals (SDG), health systems in low- and middle-income countries (LMICs) are at a critical juncture. Despite significant progress in reducing neonatal mortality in the past three decades, children are still at the highest risk of dying within the first 28 days of life [1]. Approximately 6700 neonatal deaths occurred daily in 2019, most of which were in LMICs. Currently, over 60 countries, including 90% of countries in sub-Saharan Africa, are projected to miss global SDG and Every Newborn Action Plan (ENAP) targets for neonatal mortality (≤12 neonatal deaths per 1000 live births) by 2030 [1-3]. Some global progress in preventing stillbirths has been achieved more recently, albeit slowly with vast intra- and inter-country inequities [4]. As of 2020, 56 countries were at risk of missing the ENAP stillbirth rate target (≤12 stillbirths per 1000 live births), of whom 45 need to more than double their progress to achieve the target by 2030.

The adoption and implementation of evidence-informed policies and guidelines is imperative for reaching newborn mortality and stillbirth outcomes. While encouraging the use of evidence in policymaking is a complex, multi-dimensional, and political process [5,6], it has been acknowledged as an essential practice for strengthening effective and efficient government decision-making for health [7]. Global initiatives such as the Global Strategy for Women’s, Children’s, and Adolescents’ Health (2016-2020) and ENAP have called for evidence-informed policy-making for newborn health, underlining the importance of strong health systems and policy foundations to guide programming and ultimately improve health outcomes [2,8].

Until recently, data to monitor and measure changes in newborn health policy and systems environments has been limited. Often referred to as “the bit in the middle”, policy formulation, adoption, or diffusion has been neglected in the global health literature, with striking gaps within the newborn health field [9]. Recent efforts to track newborn health policy formulation have been undertaken by Countdown to 2015 by developing tools to analyze and monitor health systems and policy change for reproductive, maternal, newborn, and child health indicators [10]. Other global initiatives have also incorporated key newborn health policy tracers within their monitoring and evaluation frameworks to track policy formulation within and across countries [2,11-13]. Since 2009, the World Health Organization’s (WHO) Maternal, Newborn, Child and Adolescent Health and Aging (MCA) and Reproductive Health and Research (RHR) departments have also undertaken a global policy survey to track country progress in adopting WHO recommendations in national health policies, strategies, and guidelines related to sexual, reproductive, maternal, newborn, child, and adolescent health (SRMNCAH) [14].

Using the most recent WHO SRMNCAH Policy Survey [14], we adapted Singh et al.’s [10] health systems and policy assessment framework to focus on the “black box” of policy inputs related to newborn health (**Figure 1**). We aimed to describe the availability of newborn health policies across the continuum of care in LMICs and to assess the relationship between LMICs’ availability of newborn health policies and their achievement of SDG and ENAP neonatal mortality and stillbirth rate targets in 2019. We hypothesized that the availability of newborn health policies may be significantly related to the achievement of SDG and ENAP neonatal mortality and stillbirth rate targets.

**Figure 1:**
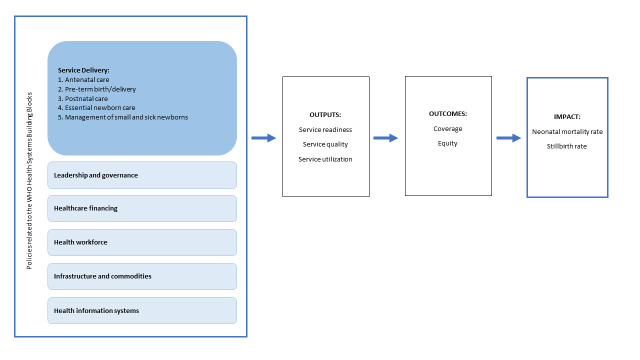
Conceptual framework.

## METHODS

We conducted a secondary analysis using the 2018-2019 WHO SRMNCAH Policy Survey indicators. In WHO’s fifth and most recent round, the 2018-2019 SRMNCAH survey was revised to align with the SDGs and Global Strategy for Women’s, Children’s and Adolescents’ Health (2016-2030) [3,8]. A SRMNCAH policy reference group was established to guide the survey’s development and expansion. Additionally, an online platform was developed to track country progress and, for the first time, collect source documentation to allow for validation of survey responses against national laws, policies, and guidelines [14].

### Key indicators and measures

**Figure 2:**
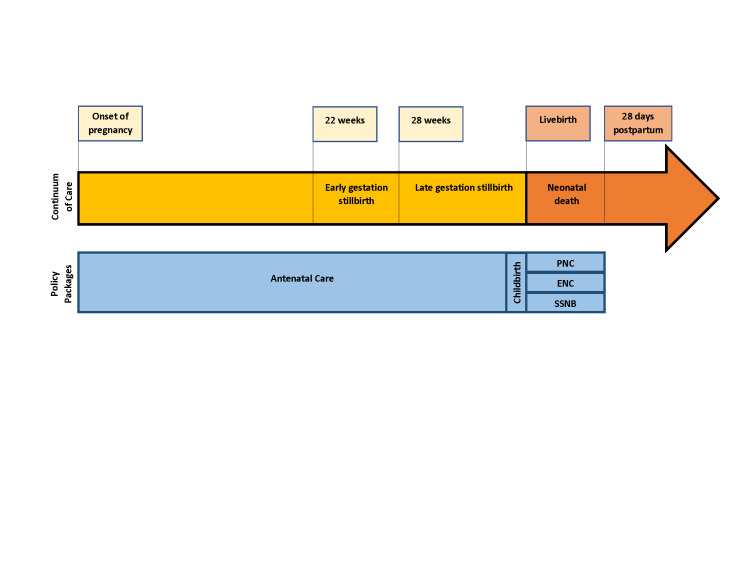
Continuum of care influencing newborn health.

We extracted key newborn health service delivery policies from the 2018-2019 SRMNCAH Policy Survey, including primary newborn health policy measures (e.g. availability of antenatal care policy) and sub-questions specifying national policy or guideline details (e.g. antenatal care (ANC) policy recommends one ultrasound scan before 24 weeks of gestation). Each measure was dichotomous, defining the availability of national policies or guidelines in each country or the lack thereof. We categorized each newborn health service delivery policy across five components of the continuum of care: ANC, childbirth, postnatal care (PNC), essential newborn care (ENC), and management of small and sick newborns (SSNB) (**Figure 2**).

We constructed composite measures to represent packages of newborn health policies for each of the five components along the continuum of care and defined the availability of a composite policy package as 1) complete (if all specified policies in the package were available, i.e. primary policy measures and related policy details), 2) partial (if at least one of the specified policies in the package was available), and 3) no availability (if none of the specified policies in the package was available). The inclusion of specific policies for each package was driven by WHO’s technical consultation and prioritization process undertaken to align and expand the 2018-2019 SRMNCAH Policy Survey content and informed through an extensive data validation exercise [14]. Consequently, the composition of each composite policy package varies in the number of specified policies.

Cross-cutting health policies that align with the WHO health system building blocks were also extracted. Maternal and newborn health policies related to governance and leadership, health workforce, infrastructure commodities, health information systems and legislative framework serve as supportive policy groundwork for which specific newborn health service delivery policies may be bolstered.

### Outcome measures

We imputed neonatal mortality and stillbirth rate estimates for 2019 from the UN Inter-agency Group for Child Mortality Estimation [1,4] and transformed them into dichotomous variables, disaggregating countries by achievement of the SDG and ENAP neonatal mortality and stillbirth rate targets. The ENAP and SDG Target 3.2 call for an end to preventable deaths of newborns with a target neonatal mortality rate (NMR) of ≤12 neonatal deaths per 1000 live births by 2030 [2,3]. The ENAP also set a target for stillbirth rates (SBR) by 2030 at ≤12 stillbirths per 1000 live births [2]. For this study, the achievement of neonatal mortality and stillbirth rate targets was defined as a mid-year 2019 NMR of ≤12 neonatal deaths per 1000 live births and SBR of ≤12 stillbirths per 1000 live births.

### Statistical analyses

We used descriptive analyses to describe the differences in the availability of newborn health service delivery policies across the continuum of care. We tested for differences across World Bank country income groups in a) newborn health policy measures and b) legislation and policies across WHO health system building blocks. We then examined the odds of achieving global NMR and SBR targets based on the availability of newborn health policy packages along the continuum of care. We assessed bivariate relationships using simple logistic regression. We estimated adjusted odds ratios (aORs), controlling for income groups and other legislation and policies across WHO health system building blocks. We assessed multi-collinearity along with goodness-to-fit using the Hosmer-Lemeshow χ^2^ goodness-to-fit test. We reported odds ratios (ORs) and 95% confidence intervals (CIs), setting *P* < 0.05 as statistically significant. We conducted all analyses using Stata 16.0 [15]. The 2018-2019 WHO SRMNCAH policy survey followed necessary WHO protocols for non-emergency, non-human-subject data collection.

## RESULTS

From 194 WHO Member States, 150 participated in the SRMNCAH Policy Survey (77%). We focused on 113 (75%) LMICs in the total sample, representing 84% of LMICs globally. **Table 1** outlines the sample distribution by WHO region and World Bank income classification. The sample’s regional representation was highest among Southeast Asia (100%) and Africa (91%) regions compared to the Western Pacific region with only 50% of LMICs represented in the sample.

**Table 1:** Characteristics of countries within the sample

Characteristic (n = 113)	n (%)
**WHO region**	
Africa	42 (37.2)
Americas	22 (19.5)
Eastern Mediterranean	13 (11.5)
Europe	15 (14.1)
Southeast Asia	11 (9.7)
Western Pacific	9 (8.0)
Income classification*	
Low income	29 (25.7)
Lower middle-income	40 (35.4)
Upper middle-income	44 (38.9)

WHO – World Health Organization

*Income classification is based on 2018 World Bank ranking.

In 2018, most LMICs had policies regarding newborn health across the continuum of care, from 96.5% of countries with ANC primary policies to 92.9% and 95.6% of countries with PNC and ENC primary policies in place, respectively (**Table 2**). However, policy specifications varied widely. Across the continuum of care, the childbirth policy package was most frequently available (85.8%; **Table 2**). In comparison, only one-fifth (22.1%) of LMICs had a SSNB policy package in place. While lower-middle and upper-middle income countries were more likely to have policy packages compared to low-income countries, only the availability of the composite ENC policy package significantly differed by World Bank income group (*P* = 0.03).

**Table 2:** Availability of key newborn health policies across the continuum of care among LMICs, by World Bank income group

	Income group, n (%)*			*P*-value†	Total
	**Low (n = 29)**	**Lower-middle (n = 40)**	**Upper-middle (n = 44)**		**(n = 113)**	
**ANC**					
1. National policies/guidelines on ANC	27 (93.1)	38 (95.0)	44 (100.0)	0.20	109 (96.5)	
*1a. ANC policy includes series of key newborn counselling and intervention topics*§	10 (34.5)	24 (60.0)	21 (47.7)	0.31	55 (48.7)	
*1b. ANC policy recommends one ultrasound scan before 24 weeks of gestation*	18 (62.1)	28 (70.0)	41 (93.2)‡	0.007	87 (77.0)	
2. National policies/guidelines on improving preterm birth outcomes	24 (82.8)	32 (80.0)	38 (86.4)	0.76	94 (83.2)	
*2a. Preterm birth policy recommends use of antenatal corticosteroids for prevention of preterm births*	22 (75.9)	30 (75.0)	36 (81.8)	0.94	88 (77.9)	
*2b. Preterm birth policy specifies clear criteria for when to use antenatal corticosteroids*	20 (69.0)	30 (75.0)	37 (84.1)	0.51	87 (77.0)	
Composite ANC policy package¶	6 (20.7)	15 (37.5)	19 (43.2)	0.23	40 (35.4)	
**Childbirth**						
1. National policy/guidelines on the right of every woman to have access to skilled care at childbirth	25 (86.2)	38 (95.0)	39 (88.6)	0.36	102 (90.3)	
2. National policy/guidelines make recommendations on the place of childbirth	24 (82.8)	37 (92.5)	36 (81.8)	0.64	97 (85.8)	
Composite childbirth policy package¶	24 (82.8)	37 (92.5)	36 (81.8)	0.65	97 (85.8)	
**PNC**						
1. National policies/guidelines on PNC for mothers and newborns	26 (89.7)	38 (95.0)	41 (93.2)	0.64	105 (92.9)	
*1a. PNC policy recommends the mother and baby rooming or being kept together until they are discharged from birthing facility*	23 (79.3)	34 (85.0)	38 (86.4)	0.86	95 (84.1)	
*1b. PNC policy recommends length of stay under observation of skilled attendant for mother and the baby after vaginal delivery at a health facility*	25 (86.2)	37 (92.5)	38 (86.4)	0.71	100 (88.5)	
*1c. PNC policy recommends postnatal follow-up contacts (visits/reviews) by a skilled attendant for mother and newborn after discharge from the facility*	26 (89.7)	37 (92.5)	37 (84.1)	0.68	100 (88.5)	
*1d. PNC policy recommends 1st contact within 24 h from birth*	24 (82.8)	34 (85.0)	28 (63.6)	0.14	86 (76.1)	
*1e. PNC policy describes who should provide care during the PNC contact(s) at home*	21 (72.4)	30 (75.0)	30 (68.2)	0.95	81 (71.7)	
*1f. PNC policy recommends assessment of the mother and newborn*	26 (89.7)	38 (95.0)	40 (90.9)	0.65	104 (92.0)	
*1g. PNC policy recommends counseling on a series of key topics*║	18 (62.1)	23 (57.5)	32 (72.7)	0.44	73 (64.6)	
Composite PNC policy package¶	10 (34.5)	17 (42.5)	17 (38.6)	0.92	44 (38.9)	
**ENC**						
1. National policy/guidelines on ENC	26 (89.7)	39 (97.5)	43 (97.7)	0.08	108 (95.6)	
*1a. ENC policy recommends immediate skin-to-skin care after birth*	26 (89.7)	37 (92.5)	41 (93.2)	0.72	104 (92.0)	
*1b. ENC policy recommends delayed cord clamping*	23 (79.3)	37 (92.5)	38 (86.4)	0.26	98 (86.7)	
*1c. ENC policy recommends early initiation of breastfeeding*	24 (82.8)	38 (95.0)	42 (95.5)	0.22	104 (92.0)	
*1d. ENC policy recommends basic resuscitation*	26 (89.7)	39 (97.5)	41 (93.2)	0.14	106 (93.8)	
*1e. ENC policy recommends hepatitis B vaccination*	19 (65.5)	36 (90.0)‡	41 (93.2)‡	0.003	96 (85.0)	
*1f. ENC policy recommends BCG vaccination*	26 (89.7)	39 (97.5)‡	38 (86.4)	0.04	103 (91.2)	
Composite ENC policy package¶	17 (58.6)	34 (85.0)‡	34 (77.3)‡	0.03	85 (75.2)	
**Management of SSNB**						
1. National policy/guidelines on management of LBW/VLBW and preterm newborns	26 (89.7)	37 (92.5)	39 (88.6)	0.58	102 (90.3)	
*1a. Policy recommends that pre-term, LBW, and VLBW newborns should be fed breastmilk*	25 (86.2)	36 (90.0)	38 (86.4)	0.70	99 (87.6)	
*1b. Policy specifies the presence of skilled personnel to assist mothers who have difficulties breastfeeding*	22 (75.9)	37 (92.5)‡	37 (84.1)	0.05	96 (85.0)	
*1c. Policy recommends Kangaroo Mother Care for clinically stable newborns weighing 2000 g or less at birth at health facilities*	24 (82.8)	31 (77.5)	31 (70.5)	0.72	86 (76.1)	
2. National standards for the management of newborn infants with severe illness	28 (96.6)	36 (90.0)	36 (81.8)	0.06	100 (88.5)	
*2a. Policy specifies availability of NICUs*	21 (72.4)	30 (75.0)	37 (84.1)	0.32	88 (77.9)	
*2b. Policy specifies availability of SNCUs*	24 (82.8)	29 (72.5)	38 (86.4)	0.10	91 (80.5)	
3. National policy/guidelines that recommends routine hemoculture before starting on antibiotics in case of suspected sepsis	12 (41.4)	20 (50.0)‡	28 (63.6)‡	0.03	60 (53.1)	
4. National policy/guideline for treatment of sick newborns with PSBI at primary health care facility when referral is not feasible	22 (75.9)	26 (65.0)	20 (45.5)‡	0.05	68 (60.2)	
Composite Management of SSNB policy package¶	3 (10.3)	7 (17.5)	15 (34.1)	0.09	25 (22.1)	

ANC – antenatal care, BCG – Bacillus Calmette-Guerin, ENC – essential newborn care, LBW – low birth weight, NICU – neonatal intensive care units, PNC – postnatal care, PSBI – possible serious bacterial infection, SNCU – special newborn care units, VLBW – very low birth weight, g – grams, h – hours

*Income classification is based on 2018 World Bank ranking.

†We calculated *P*-values using a χ^2^ test to assess differences across levels and employed post hoc testing to compare lower-middle and upper-middle-income countries to low-income countries (reference group).

‡Statistically different from the reference group.

§ANC topics include birth preparedness and complication readiness, nutrition during pregnancy, iron and folic acid during pregnancy, immunization during pregnancy, screening for sexually transmitted infections, prevention and treatment of human immunodeficiency virus (HIV) in pregnancy, prevention and treatment of syphilis in pregnancy, prevention of tuberculosis, prevention and management of gestational diabetes, counselling on tobacco, alcohol, and substance abuse during pregnancy, and partner involvement and counselling.

¶We defined composite policy packages as having all outlined policy components available.

║PNC topics include breastfeeding, nutrition, exercise and rest, family planning, recognition and reporting of illness/sickness for the mother and the newborn, well-being advice for the mother, and newborn early childhood development.

**Table 3** outlines the availability of legislation and policies related to newborn health service provision by World Bank income group. Overall, the availability of policies across the WHO health system building blocks was high (i.e. >70%), with the exception of national policies that require stillbirths to be reviewed, available in only 43.4% of LMICs. The availability of these supportive legislation and health systems policies did not vary by income group.

**Table 3:** Availability of legislation and policies across WHO health system building blocks among LMICs, by World Bank income group

	Income group, n (%)*			*P*-value†	Total
	**Low (n = 29)**	**Lower-middle (n = 40)**	**Upper-middle (n = 44)**		**(n = 113)**	
**Leadership and governance**					
National law that guarantees universal access to primary health care	19 (65.5)	33 (82.5)	37 (84.1)	0.37	89 (78.8)	
National policy/guideline to improve QoC for health services for newborn health	22 (75.9)	34 (87.2)	37 (84.1)	0.49	93 (83.0)	
National QoC standards and protocols for delivery of services in health facilities for newborns	22 (75.9)	31 (81.6)	30 (73.2)	0.61	83 (76.9)	
**Healthcare financing**						
National law/policy on free access to health services in the public sector at the point of use for newborns	25 (86.2)	35 (87.5)	40 (90.9)	0.50	100 (88.5)	
**Health workforce**						
National policies/guidelines that set forth a competency framework for maternal and/or newborn health care	22 (75.9)	32 (80.0)	34 (77.3)	0.71	88 (77.9)	
Continuous professional education system in place for primary health care clinicians and/or nurses to receive maternal and/or newborn-specific training	21 (72.4)	31 (77.5)‡	28 (63.6)	0.05	80 (70.8)	
Task-shifting policy allowing nurse, midwife, nurse-midwife, or medical assistant to conduct newborn health interventions§	22 (75.9)	30 (75.0)	30 (68.2)	0.90	82 (72.6)	
**Infrastructure and commodities**						
National policy/guideline on availability of clean water and sanitation for deliveries	26 (89.7)	31 (77.5)	35 (79.5)	0.65	92 (81.4)	
National essential medicines list includes injectable antibiotics for newborn sepsis	29 (100.0)	37 (92.5)	41 (93.2)	0.26	107 (94.7)	
National essential medicines list includes antenatal corticosteroids	27 (93.1)	37 (92.5)	40 (90.9)	0.42	104 (92.0)	
National list of commodities includes newborn resuscitation devices	23 (79.3)	32 (80.0)	38 (86.4)	0.76	93 (82.3)	
National list of commodities includes oxygen supply	26 (89.7)	32 (80.0)	38 (86.4)	0.54	96 (85.0)	
**Health information systems**						
National policy/guideline/law that requires neonatal deaths to be reviewed	19 (65.5)	30 (75.0)	33 (75.0)	0.75	82 (72.6)	
National policy/guideline/law that requires stillbirths (fresh or macerated) to be reviewed	10 (34.5)	19 (47.5)	20 (45.5)	0.66	49 (43.4)	

QoC – quality of care

*Income classification is based on 2018 World Bank ranking.

†We calculated *P*-values using a χ^2^ test to assess differences across levels and employed post hoc testing to compare category levels.

‡Statistically different from the reference group (upper-middle income).

§Newborn health interventions include newborn resuscitation, kangaroo mother care, care for sick infants, or lactating support.

**Figure 3:**
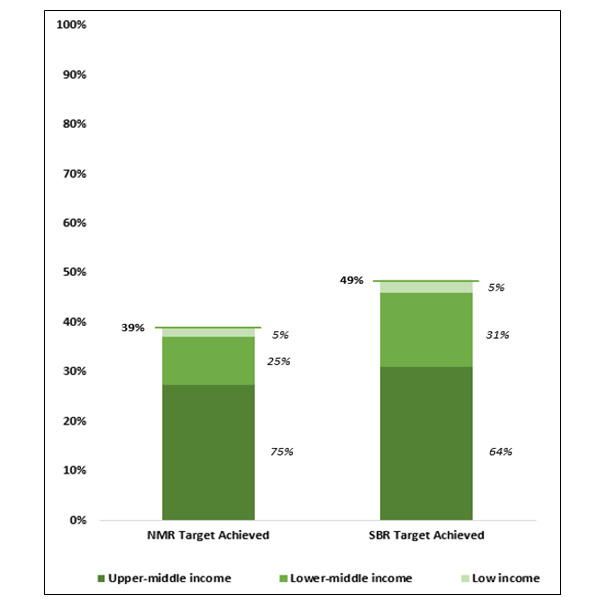
Achievement of Global SDG and ENAP neonatal mortality and stillbirth rate targets by 2019 among LMICs, disaggregated by World Bank Income classification (%).

By 2019, 39% of LMICs achieved the global SDG and ENAP NMR target (**Figure 3**). Countries achieving NMR targets varied by income level: 75% were upper-middle income, 25% were lower-middle income, and 5% were classified as low-income countries. Similarly, 49% of LMICs achieved the global ENAP SBR target, of which only 5% comprised low-income countries.

**Figure 4:**
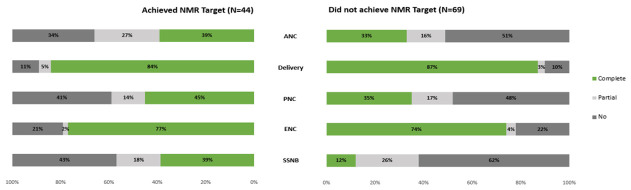
Proportion of LMICs with composite policy packages, comparison between countries that have and have not achieved the SDG/ENAP NMR target by 2019.

The availability of composite ANC, childbirth, PNC, and ENC policy packages was not associated with having achieved the SDG and ENAP NMR target by 2019 (**Table 4**). However, countries with policy packages on the management of SSNB were 4.81 times more likely to have reached the global NMR target (95% CI = 1.77-13.06) compared to those with partial packages or no related policies in place (**Table 4**). The relationship remained significant (*P* = 0.04) when controlling for income group and other related legislation and policies across WHO health system building blocks (aOR = 4.40; 95% CI = 1.09-17.79). **Figure 4** depicts the differences in availability of each of the composite policy packages along the continuum of care by achievement of the global SDG and ENAP NMR targets. While the proportion of LMICs with a complete ANC, childbirth, PNC, and ENC policy package in place was similar between NMR target achievers and non-achievers, the SSNB policy package was available in 39% of LMICs that had achieved the global NMR target compared to only 12% of those who have not yet reached an NMR of ≤12 neonatal deaths per 1000 live births. We found no relationship between the achievement of the ENAP SBR target and the availability of the ANC policy package (*P* = 0.77).

**Table 4:** Odds of having achieved established global neonatal mortality rate targets by 2019, given the availability of newborn health policy packages

Availability of composite newborn health policy packages	Model 1*		Model 2†	
**OR (95% CI)**	***P*-value**	**aOR (95% CI)**	***P*-value**
**NMR target achieved‡**				
ANC	1.72 (0.72-4.12)	0.22	1.18 (0.31-4.52)	0.81
Childbirth	0.86 (0.26-2.92)	0.81	1.20 (0.08-16.92)	0.89
PNC	1.52 (0.67-3.49)	0.31	1.74 (0.46-6.59)	0.42
ENC	1.11 (0.44-2.83)	0.83	0.93 (0.18-4.85)	0.93
Management of SSNB	4.81 (1.77-13.06)	0.002	4.40 (1.09-17.79)	0.04
**SBR target achieved§**				
ANC	1.50 (0.65-3.47)	0.34	1.24 (0.29-5.28)	0.77

aOR – adjusted odds ratio, CI – confidence ratio, ANC – antenatal care, ENC – essential newborn care, NMR – neonatal mortality rate, OR – odds ratio, PNC – postnatal care, SBR – stillbirth rate

*Model 1 estimates the crude odds of achieving NMR and SBR targets between countries with and without ANC, childbirth, PNC, ENC, and management of small and sick newborns policy packages available.

†Model 2 adjusts the odds of achieving NMR and SBR targets between countries with and without each composite newborn health policy package by controlling for income groups and policies corresponding with each WHO health system building block. Model 2 for achievement of NMR targets excludes “National policy/guideline/law that requires stillbirths (fresh or macerated) to be reviewed” covariate. Model 2 for achievement of SBR targets excludes the following covariates: national essential medicines list includes injectable antibiotics for newborn sepsis, national list of commodities includes newborn resuscitation devices, national list of commodities includes oxygen supply, and national policy/guideline/law that requires neonatal deaths to be reviewed.

‡NMR target achieved defined as a mid-year 2019 neonatal mortality rate ≤12 per 1000 live births, as indicated in the Sustainable Development Goals and Every Newborn Action Plan.

§We defined SBR target achieved as a mid-year 2019 stillbirth rate ≤12 per 1000 live births, as indicated in the Every Newborn Action Plan.

## DISCUSSION

Understanding the policy landscape related to newborn care provides important insights regarding the availability of newborn health services in LMICs. We based our study on other efforts tracking key newborn health policy tracers by assessing the availability of composite policy packages, which consider essential evidence-based policy specifications related to service delivery models and interventions proven to improve newborn health outcomes. With the exception of childbirth and ENC policies, we found fairly low availability of composite policy packages across the continuum of care. We hypothesize that high availability of the childbirth policy package (85.8%) is driven by its inherent construction (i.e. fewer specified components compared to other policy packages) and due to the recent global prioritization of attaining universal access to skilled birth attendance and institutional delivery [16]. Similarly, credit for the availability of the ENC policy package in over 75% of LMICs could be attributed to the shift in global agendas in the early 2000s to prioritize newborn survival [17]. A multitude of stakeholders have since translated evidence on the components of essential newborn care (warmth, immediate skin-to-skin care, early breastfeeding, umbilical cord care, eye care, vitamin K administration, and immunization) into policy and practice. Supporting these efforts, the WHO developed an essential newborn care course in 2010, with a second edition set to be launched in 2022 [18].

Evidence of the relationship between the achievement of the global NMR target and the availability of the composite policy package related to the management of SSNB suggests that policies related to specialized care in the antepartum and immediate postpartum period are critical for neonatal survival. The primary causes of death for neonates include complications from preterm birth, intrapartum-related conditions (i.e. birth asphyxia), and infection, with small and sick newborns facing the highest risk of death [19-21]. Some policy specifications of the SSNB policy package included recommendations on provision of Kangaroo Mother Care for low birthweight newborns, availability of neonatal intensive care units, and recommendations for routine hemoculture before starting on antibiotics, in case of suspected sepsis – all of which are directly related to the key drivers of neonatal mortality.

However, despite the 4-fold odds of achieving the global NMR targets when the SSNB policy package is employed, only 22% of LMICs reported having the SSNB policy package. This could be related to weakened health system capacity driving countries to focus on essential newborn care until more advanced and complex newborn health interventions for SSNB are feasible to implement at scale. Moxon et al. [22] outlined the extensive health system bottlenecks that impede the provision of timely, high-quality inpatient care that SSNBs need to survive, namely human resources for health (i.e. the need for a neonatal nursing cadre) and health financing for sustained funding and insurance schemes to cover inpatient care. Nonetheless, as the adoption and implementation of ENC policies and guidelines rapidly expands and health systems strengthen, more and more LMICs will be ready to adopt and implement evidence-based policies for inpatient care for SSNB. With the release of WHO Standards for Improving Quality of Care for Small and Sick Newborns in 2020, health systems and policy environments will need to evolve to support new guidance on the key components of care for SSNBs [23]. Subsequent WHO SRMNCAH Policy Survey rounds should consider these new guidelines to enable close tracking of critical evidence-based policies related to specialized care for SSNBs.

Although we found a significant association between policy enactment and achievement of newborn health outcome targets, assessing the causal pathway from policy to practice to impact is complex. Evidence-informed health policy formulation is often not sufficient to reduce newborn mortality or stillbirth rates, as socio-economic, political, and health system determinants influence policy adoption and implementation of key newborn health interventions [24,25]. Consequently, our analysis is limited, accounting only for the supporting health policy environment and country income group, since contextual factors and other newborn health indicators along the causal pathway were not systematically available for most LMICs in the sample. This created unexplained variability within our analyses, as can be seen from the wider confidence intervals (**Table 4**). Another limitation is the differences between composite policy packages. For this study, we constructed composite policy packages based on available data, which represent analytical packages rather than comprehensive conceptual policy packages related to newborn health service delivery. To expand and align the 2018-2019 SRMNCAH policy survey with key global strategies, WHO established an expert SRMNCAH policy reference group to identify priority policy areas and guide survey development. Based on expert consultation, the final survey had ANC, childbirth, PNC, ENC, management of premature or low birthweight newborns, and management of sick newborn policy sections within the maternal and newborn health module. We constructed the composite policy packages based on these policy sections, extracting key policies and their specifications related to newborn health. Consequently, the number of policies included in each package varied from two (childbirth policy package) to seven (PNC and SSNB policy packages), which may have affected our associations of interest among LMICs. Furthermore, the data collected in the SRMNCAH survey are self-reported by country representatives (i.e. Ministry of Health or UN agencies) and may have variable quality, despite validation efforts to verify information reported against publicly available data [14].

## CONCLUSIONS

We presented a comprehensive view of the newborn health policy landscape in LMICs. While general policies related to newborn health were adopted in most countries, evidence-based policy specifications were not in place or detailed in many contexts. In particular, we found that the adoption of policies related to the management of small and sick newborns may affect the availability of and access to critical inpatient care for the newborns at greatest risk of death. Given current trajectories of neonatal mortality in LMICs, there is a dire need for supportive health systems and policy environments for newborn health across the continuum of care. Adoption and implementation of evidence-informed newborn health policies will be crucial for putting LMICs on track to meet global SDG and ENAP newborn and stillbirth targets by 2030.
